# Identifying maternal and infant factors associated with newborn size in rural Bangladesh by partial least squares (PLS) regression analysis

**DOI:** 10.1371/journal.pone.0189677

**Published:** 2017-12-20

**Authors:** Alamgir Kabir, Md. Jahanur Rahman, Abu Ahmed Shamim, Rolf D. W. Klemm, Alain B. Labrique, Mahbubur Rashid, Parul Christian, Keith P. West

**Affiliations:** 1 Department of Statistics, University of Rajshahi, Rajshahi, Bangladesh; 2 The JiVitA Maternal and Child Health and Nutrition Research Project of Johns Hopkins University, Gaibandha, Bangladesh; 3 Helen Keller International, Dhaka, Bangladesh; 4 Center for Human Nutrition, Department of International Health, Bloomberg School of Public Health, Johns Hopkins University, Baltimore, Maryland, United States of America; 5 Helen Keller International, New York, New York, United States of America; 6 The Bill & Melinda Gates Foundation, Seattle, Washington, United States of America; BRAC, BANGLADESH

## Abstract

Birth weight, length and circumferences of the head, chest and arm are key measures of newborn size and health in developing countries. We assessed maternal socio-demographic factors associated with multiple measures of newborn size in a large rural population in Bangladesh using partial least squares (PLS) regression method. PLS regression, combining features from principal component analysis and multiple linear regression, is a multivariate technique with an ability to handle multicollinearity while simultaneously handling multiple dependent variables. We analyzed maternal and infant data from singletons (n = 14,506) born during a double-masked, cluster-randomized, placebo-controlled maternal vitamin A or β-carotene supplementation trial in rural northwest Bangladesh. PLS regression results identified numerous maternal factors (parity, age, early pregnancy MUAC, living standard index, years of education, number of antenatal care visits, preterm delivery and infant sex) significantly (p<0.001) associated with newborn size. Among them, preterm delivery had the largest negative influence on newborn size (Standardized β = -0.29 − -0.19; p<0.001). Scatter plots of the scores of first two PLS components also revealed an interaction between newborn sex and preterm delivery on birth size. PLS regression was found to be more parsimonious than both ordinary least squares regression and principal component regression. It also provided more stable estimates than the ordinary least squares regression and provided the effect measure of the covariates with greater accuracy as it accounts for the correlation among the covariates and outcomes. Therefore, PLS regression is recommended when either there are multiple outcome measurements in the same study, or the covariates are correlated, or both situations exist in a dataset.

## Introduction

In developing countries, small size at birth is common, reflecting combined effects of inadequate intrauterine growth and preterm birth. Both birth conditions are associated with increased risks of poor postnatal growth [1] and infant mortality [2], and diverse maternal nutritional, health and socioeconomic factors [3–5]. Although weight is most commonly measured at birth, other measurements, including length and circumferences of the head, chest and arm provide additional information on newborn size and proportionality, as well as confirmatory and novel insights about fetal growth [2]. While maternal health and socioeconomic status have been shown to be associated with risk of small birth size in low resource settings in South Asia [3,5], there remains a need to adapt analytical techniques to more efficiently and simultaneously explore risk factors of birth size to identify pregnant women who may benefit from antenatal, obstetric and postnatal care. In this paper, we illustrate the use of partial least squares (PLS) regression analysis to quantify associations between materno-infant and household socioeconomic characteristics and multiple measures of newborn size, drawing on a large population cohort of infants whose mothers participated in a maternal vitamin A and beta-carotene supplementation in rural, northern Bangladesh [6].

In exploring associations between health outcomes and multiple risk factors, observational epidemiologic studies often deal with data that include both a set of exposure variables and a set of outcome variables. Frequently, the variables are interrelated to some extent and consequently, multicollinearity often exists. Routine statistical approaches such as multiple linear regression or principal component regression (PCR) are usually challenged with multiple testing. Specifically, using separate statistical significance tests for each regression equation when there are multiple outcomes substantially increases the risk of Type 1 error [7]. Additionally, the multiple linear or PCR analysis is not able to account for the correlation structure among the dependent variables. A multivariate regression approach called partial least squares (PLS) regression developed by Harman Wold in the 1960’s [8,9], which can simultaneously consider more than one dependent variables, can address the correlation structure among the dependent variables, and also efficiently handle multicollineary. Thus, we propose through this study that PLS regression has the potential to be a useful method to predict multiple facets of a health outcome—in this instance, infant size at birth from maternal, infant and other household factors in a rural South Asian population [6]. We also compare the performance of PLS regression with PCR and the individual predictive ability of each these two methods.

## Materials and methods

### Ethics statement

The overall Jivita study protocol was reviewed and approved by both the Bangladesh Medical Research Council (BMRC) and the Institutional Review Board (IRB) of Johns Hopkins Bloomberg School of Public Health, Baltimore, Maryland, USA. Documented consent was given by all participating women.

In this study, PLS regression was used to assess the effect of maternal and infant factors on newborn size at birth. The data reported in this analysis were collected during a field based study assessing the efficacy of weekly antenatal vitamin A or β-carotene versus placebo supplementation on maternal and infant mortality through 6 months of age from July 2001 to January 2007. Details of this study conducted in a contiguous ~435 sq km area in rural northwestern Gaibandha and Rangpur Districts of Bangladesh with a population of ~650,000 are available elsewhere [6,10].

On enrollment into the intervention trial, consenting mothers were interviewed in the 1^st^ trimester about household socioeconomic condition, education, demographic characteristics, previous pregnancy history, recent morbidity history and 7-day food intake and mid-upper arm circumference. A Living Standard Index (LSI) was constructed using principal component analysis from household socio-economic variables and was used to describe socio-economic status [11]. At 3 months postpartum another interview was completed to collect dietary, morbidity, antenatal care (ANC) received, and events and care received during labor and delivery, among other postpartum factors. Birth size measurements were added as part of a newborn, placebo-controlled, single-dose vitamin A supplementation trial that was initiated among infants born during the 2^nd^ half of the maternal trial [12]. Shortly after birth, newborns were dosed with allocated supplements and, shortly thereafter [median age (IQR): 18 (9, 36) hours of age], one of 56 standardized, female anthropometrists measured newborn weight, length, and mid-upper arm, head, and chest circumferences. Birth weight was measured to the nearest 10 g using a Tanita BD-585 digital pediatric scale (Tanita Corporation, Tokyo, Japan). Length was measured to the nearest 0.1 cm using an affixed headboard and movable footplate that had been fashioned for use with the Tanita scale. Circumferential measurements were made to the nearest 0.1 cm with a Ross insertion tape (Abbott Laboratories, Columbus, OH). All measurements, except for weight, were measured in triplicate following standard methods [13]. Small infant size was defined by a birth weight <2.5 kg, MUAC <10 cm, head circumference <33 cm or chest circumference < 30.5 cm [14]. Singleton live born infants measured within 72 hours of birth (n = 16,290, 75% of 21,585 singleton live born infants) were eligible, out of which 14,506 (89% of 16,290) had complete data and were included in this analysis.

The maternal characteristics included in this study were age at enrollment, parity, mid upper arm circumference (cm), education (yrs), living standard index, number of antenatal care visits, and maternal supplementation group. Additional infant characteristics included preterm (<37 week of gestation) delivery and infant sex (female/male).

### Partial least squares regression

The classical regression methods usually meet four main challenges with: (i) a large number of variables, (ii) correlated predictors, (iii) smaller sample size with a large number of variables and (iv) having more than one response variables simultaneously [15]. To overcome these problems, the researchers usually take some measures; they may remove some variables [16] or may use multivariate reduction techniques like principal component analysis to reduce the multidimensionality in the predictor or response variables [17]. However, removing variables may often incur selection of redundant variables which have no significant effect on the response variable. On the other hand, despite the fact that the dimensionality reduction techniques reduce the number of predictors by using latent variables instead, the latent variables are usually derived by maximizing the covariation among the predictors instead of maximizing the covariation among the response variables. Consequently, this may produce patterns or syndromes within the predictor variables making little or no biological sense [15]. The appropriate solution of these challenges is using PLS regression [15].

Although PLS regression is comparatively new, its use in research is gradually increasing. The great strength of PLS regression is parsimony [18]. Initially, used in analytic chemistry [19–21], PLS now it is gaining popularity in public health [22–24], bioinformatics [25], ecology [15,26] and agriculture [27]. As it is computationally much more intensive, the advent of statistical packages such as, R, SAS, STATA, MatLab and STATISTICA also facilitates its wider application.

Similar to principal component regression (PCR), PLS regression analysis is a data-dimension reduction method that extracts a set of orthogonal factors called latent variables which are used as predictors in the regression model [9]. The major difference with PCR is that principal components are determined solely by the X variables, whereas with PLS, both the X and Y variables influence the construction of latent variables. The intention of PLS is to form components (latent variables) that capture most of the information in the X variables that is useful for predicting Y variables, while reducing the dimensionality of the regression problem by using fewer components than the number of X variables. PLS is considered especially useful for constructing prediction equations when there are many explanatory variables and comparatively little sample data [28].

The PLS regression identifies the latent variables stored in matrix T and they model X and predict Y simultaneously. Then the following expression can be written as,

$$X=TP^{T}\;and\;\hat{Y}=TBC^{T}$$ (1)

Where, P and C are loadings and B is diagonal matrix. These latent variables are ordered according to the variance of Ŷ they explain. Ŷ can also be written as

$$\hat{Y}=TBC^{T}=XB_{PLS,}where\;B_{PLS}=P^{T+}BC^{T}$$ (2)

**P**^**T+**^ is the Moore-Penrose pseudo-inverse of P^T^. The matrix B_PLS_ has J rows and K columns and is equivalent to the regression weights of multiple regression.

The latent variables are computed iteratively using Singular Value Decomposition (SVD). In each iteration, SVD constructs orthogonal latent variables for X and Y and corresponding regression weights [9]. The algorithm for PLS regression is as follows:

Step 1: Transform X and Y into Z-scores and store in matrices X_0_ and Y_0_
Step 2: Compute the correlation matrix between X_0_ and Y_0_, R_1_ = X_0_^T^Y_0_
Step 3: Perform singular value decomposition (SVD) on R_1_ and produce two sets of orthogonal singular vectors w_1_ and c_1_ corresponding to the largest singular value, λ_1_.
Step 4: The first latent variable for X is given by T_1_ = X_0_^T^w_1_.
Step 5: Normalize T_1_ such that T_1_^T^T_1_ = 1
Step 6: The loadings of X_0_ on T_1_ is computed as
  $$P_{1}=X_{0}^{T}T_{1}\;and\;{\hat{X}}_{1}=T_{1}^{T}P_{1}.$$
Step 7: Compute U_1_ = Y_0_c_1_ *and*
  $${\hat{Y}}_{1}=U_{1}c_{1}^{T}=T_{1}b_{1}c_{1}^{T},\;where,\;b_{1}=T_{1}^{T}U_{1}$$ (3)
  The scalar *b*_*1*_ is the slope of the regression of Ŷ on T_1_. Eq (3) shows that Ŷ is obtained as linear regression from the latent variable extracted from X_0_. Matrices ${\hat{X}}_{1}$ and Ŷ_1_ are then subtracted from the original X_0_ and Y_0_ respectively to give deflated X_1_ and Y_1_.
Step 8: Compute the input matrices for the next iteration,
  $$X_{1}=X_{0}-{\hat{X}}_{1}and\;Y_{1}=Y_{0}-{\hat{X}}_{1}$$
Step 9: The first set of latent variables has now been extracted. Now perform SVD on R_2_ = X_1_^T^ Y_1_ we get w_2_, c_2_, T_2_ and b_2_ and the new deflated matrices X_2_ and Y_2_.
Step 10: The iterative process continues until X is completely decomposed in to L components (where L is the rank of X). When this is done, the weights (i.e., all the w’s) for x are stored in the J by L matrix W (whose l-th column is w_l_).

The latent variables of X are stored in matrix T, the weights for Y are stored in C, the latent variables of Y are stored in matrix U, the loadings for X are stored in matrix P and the regression weights are stored in a diagonal matrix B. The regression weights are used to predict Y from X.

Now the question is how many components, or t’s will have to be retained in the final model. The answer can be obtained by comparing the cross validation Root-Mean Squared Error of Prediction (RMSEP) for different number of components. The component at which the cross validation RMSEP has a meaningful change is used in the final model. To choose the optimum number of components for both PLS and principal component regression, root mean squared error of prediction (RMSEP) were calculated using different number of components. We performed approximate t-tests of regression coefficients based on jackknife variance estimates [29].

We constructed a correlation plot of the variables to observe how variables are correlated with each other and also between the birth size variables and maternal variables. The closer a variable appears to the perimeter of the circle, the better it is represented, and if two variables are highly correlated they appear near each other. If two variables are negatively correlated they will tend to appear in opposite extremes. If two variables are uncorrelated, they will be orthogonal to each other. We plotted the scores of first two components, t_1_ vs t_2_, which helped us to assess if there is any natural grouping or interactions among variables.

To examine the advantage of PLS regression over principal component regression, we calculated Pearson’s correlation coefficients between the predicated values (by PLS and principal component regression with 1 to 5 components respectively) and the observed values of infant’s size variables. This correlation coefficient indicate the predicative power of the model: if the model has perfect predictive ability then the correlation coefficient will be 1. So, the more the correlation coefficient, the higher the predictive power of a given model is. For this analysis, we used the R packages: “plsdepot”, “pls” and “mixOmics”.

## Results

More than half of the infants were small at birth. More than 50% mother had parity of at least 1, mean (SD) maternal age was 21.96 (5.88) yrs and maternal early pregnancy MUAC was 22.99 (1.97) cm. About 75% mothers reported not having had any ANC visits throughout the pregnancy. Preterm delivery was 27% and 51% infants were male (Table 1).

**Table 1:** Descriptive statistics for birth size and maternal socio-demographic factors from rural North West Bangladesh in 2002–2007, n = 14506.

Variables	Mean (SD)/ n (%)	Median (IQR)
**Birth size, within 72 hours of birth**		
Weight, kg	2.44 (0.42)	2.44 (2.18, 2.71)
Length, cm	46.43 (2.41)	46.50 (45.10, 48.00)
MUAC, cm	9.31 (0.84)	9.30 (8.80, 9.90)
HC, cm	32.36 (1.63)	32.50 (31.40, 33.40)
CC, cm	30.40 (2.09)	30.50 (29.20, 31.70)
**Maternal and infant factors**		
Parity	1.18 (1.41)	1.00 (0.00, 2.00)
Age at enrollment, year	21.96 (5.88)	21.00 (17.00, 26.00)
Early pregnancy MUAC, cm	22.99 (1.97)	22.90 (21.60, 24.10)
Living Standard Index (LSI)	0.08 (0.96)	−0.11 (−0.65, 0.67)
Years of education	3.84 (3.86)	3.00 (0.00, 7.00)
No. of ANC visit	0.52 (1.15)	0.00 (0.00, 1.00)
Vitamin A supplementation^†^	4897 (33.76)	-
β-carotene supplementation^†^	4803 (33.11)	-
Preterm delivery^†^	3904 (26.91)	-
Male infant^†^	7384 (50.90)	-

^†^ Dichotomous variables are presented as n(%)

**Fig 1:**
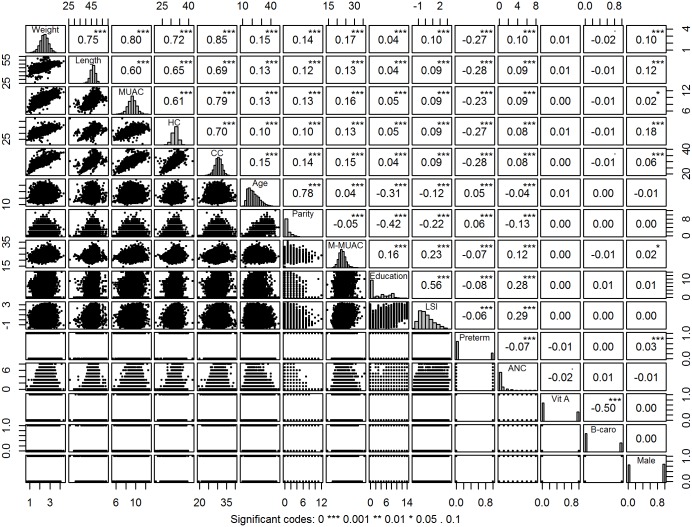
Pair wise correlation and scatter plot matrix of the study variables. (MUAC, Mid-Upper Arm Circumference; HC, Head circumference; CC, Chest Circumference; M-MUAC, Maternal MUAC; LSI, Living Standard Index; ANC, Antenatal care visit; Vit A, Maternal vitamin A supplementation; B-care, Maternal β-carotene supplementation; Male, Infant’s sex as male).

Fig 1 simultaneously displays the correlation between variables (superdiagonal), two-way scatter plot (subdiagnal), and the histogram of each variable (diagonal). All the birth size variables were significantly (p<0.001) highly correlated with each other. All the predictors except vitamin A and β-carotene supplementation were significantly (p<0.05) correlated with all the birth size variables. Among them, preterm delivery had highest negative correlation with all the birth size variables (|r|>0.20, p<0.001) and maternal age, parity and MUAC had higher correlation compared to other variables (r≥0.10, p<0.001). Predictors were also correlated with each other, maternal age was highly correlated with parity (r = 0.78, p<0.001), maternal education was moderately positively correlated with LSI (r = 0.56, p<0.001) and negatively correlated with parity (r = -0.42, p<0.001) and age (r = -0.31, p<0.001).

**Fig 2:**
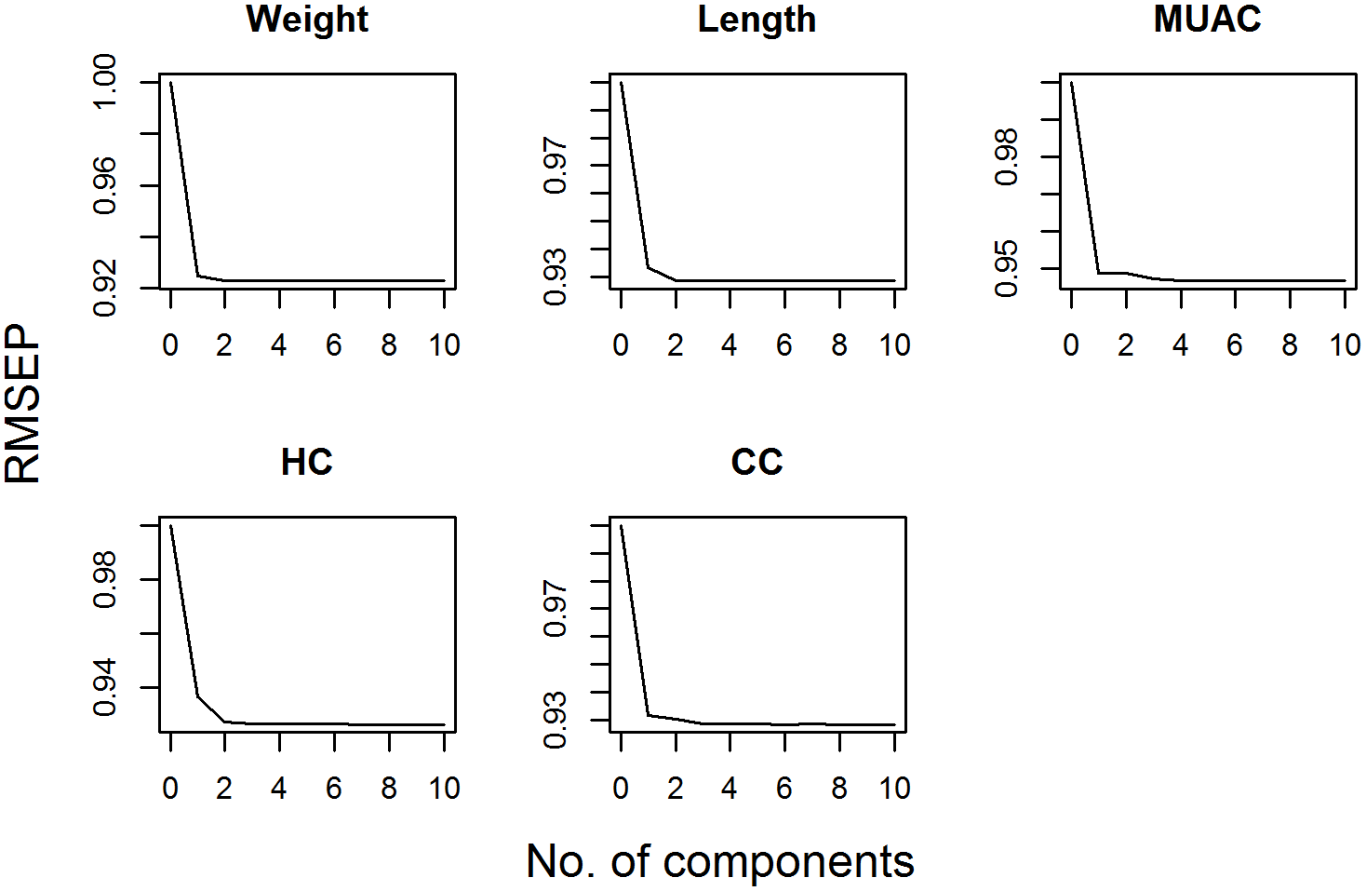
Root mean squared error of prediction (RMSEP) for different number of components of partial least squares (PLS) repression.

**Fig 3:**
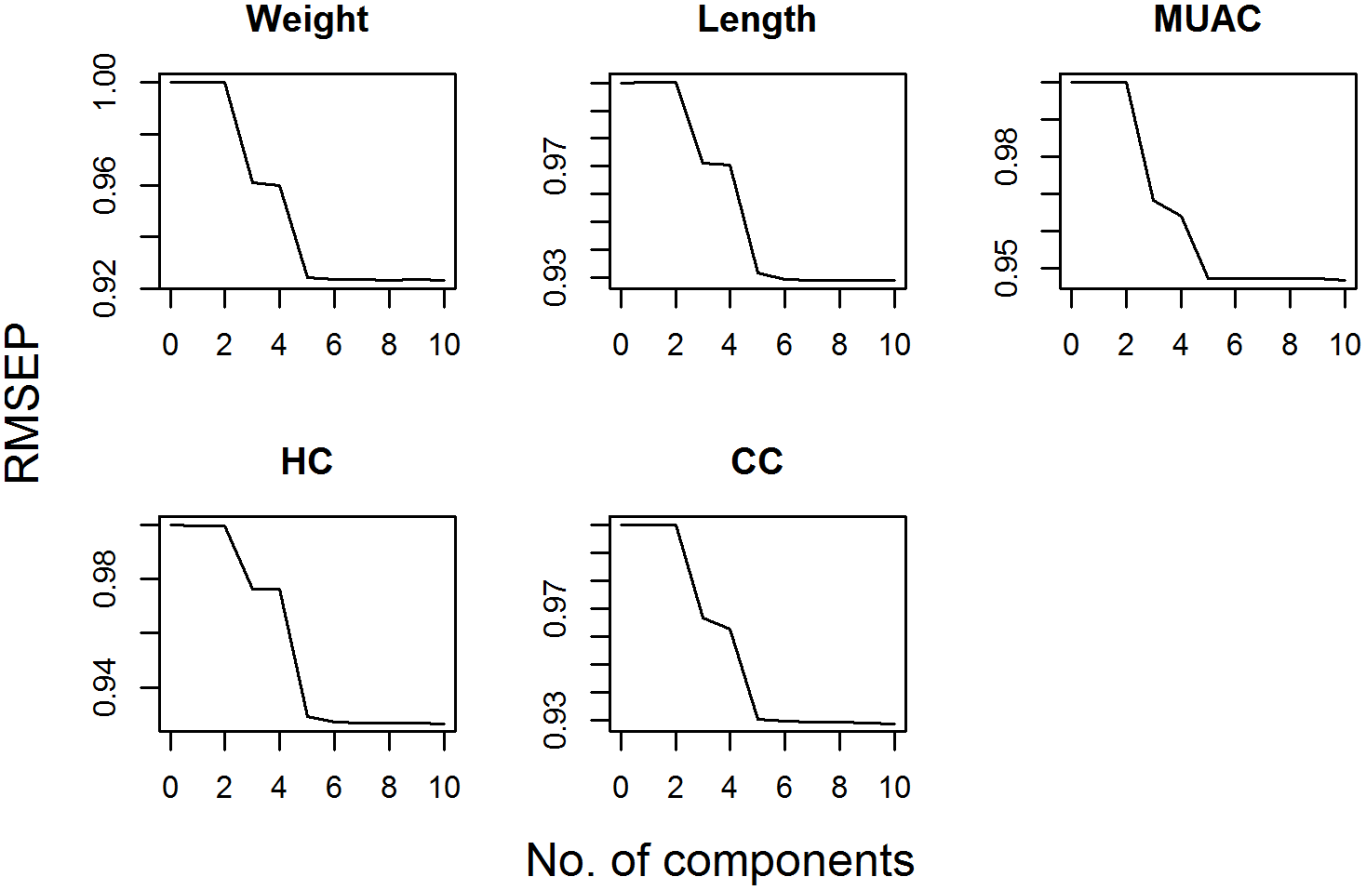
Root mean squared error of prediction (RMSEP) for different number of components of principal component regression (PCR).

The RMSEP was plotted against number of components used in the PLS regression model and principal component regression models in Figs 2 and 3 respectively. These figures suggested that 2 components would be included in the PLS regression model while 5 components would be included in PCR.

Table 2 presents the standardized PLS regression coefficient with 2 components. Except the prenatal supplementation of vitamin A and β-carotene, all the variables had significant (p <0.001) association with infant’s size at birth. Preterm delivery was the most influential variable, which was negatively associated with infant’s size at birth (standardized β = -0.27, -0.27, -0.19, -0.29, and -0.25 for weight, length, MUAC, HC and CC, respectively) followed by infant’s sex and maternal parity, MUAC, and age.

**Table 2:** Standardized PLS regression coefficients using 2 components with Jackknife SE and p-value to predict infant’s size at birth from maternal factors.

Maternal and infant factors	Weight		Length		MUAC		HC		CC	
	β (SE)	p-value	β (SE)	p-value	β (SE)	p-value	β (SE)	p-value	β (SE)	p-value
Age	0.10 (0.01)	<0.001	0.09 (0.00)	<0.001	0.10 (0.01)	<0.001	0.07 (0.01)	<0.001	0.10 (0.01)	<0.001
Parity	0.11 (0.00)	<0.001	0.10 (0.01)	<0.001	0.09 (0.01)	<0.001	0.09 (0.01)	<0.001	0.10 (0.00)	<0.001
Early pregnancy MUAC	0.11 (0.01)	<0.001	0.10 (0.01)	<0.001	0.11 (0.01)	<0.001	0.09 (0.01)	<0.001	0.11 (0.01)	<0.001
Education	0.03 (0.00)	<0.001	0.03 (0.01)	<0.001	0.03 (0.01)	0.001	0.02 (0.01)	0.009	0.03 (0.00)	<0.001
LSI	0.06 (0.00)	<0.001	0.05 (0.01)	<0.001	0.07 (0.01)	<0.001	0.03 (0.01)	<0.001	0.06 (0.00)	<0.001
Preterm	-0.27 (0.01)	<0.001	-0.27 (0.01)	<0.001	-0.19 (0.01)	<0.001	-0.29 (0.01)	<0.001	-0.25 (0.01)	<0.001
No of ANC visit	0.06 (0.01)	<0.001	0.05 (0.00)	<0.001	0.07 (0.01)	<0.001	0.04 (0.01)	0.001	0.06 (0.01)	<0.001
Vitamin A sup	0.01 (0.01)	0.467	0.00 (0.01)	0.592	0.01 (0.01)	0.371	0.00 (0.01)	0.733	0.00 (0.01)	0.468
β-carotene sup	-0.01 (0.01)	0.178	-0.01 (0.01)	0.224	-0.01 (0.01)	0.142	-0.01 (0.01)	0.300	-0.01 (0.01)	0.176
Male infant	0.12 (0.01)	<0.001	0.12 (0.01)	<0.001	0.07 (0.01)	<0.001	0.16 (0.01)	0.001	0.11 (0.01)	<0.001
R^2^	0.15	--	0.14	--	0.10	--	0.14	--	0.14	--

Abbreviations: SE, Standard Error; MUAC, Mid-Upper Arm Circumference; LSI, Living Standard Index; Mom MUAC, Maternal Mid-Upper Arm Circumference.

Some of the coefficients estimated using the ordinary linear regression (LR) were different compared to the coefficients estimated using PLS regression (S1 Table). Coefficients of maternal age estimated using PLS regression were continually higher than the coefficients estimated using LR. Conversely, coefficients of parity as well as maternal education estimated using PLS regression were lower than their respective coefficients estimated using LR. However, in case of LSI, the coefficients were arbitrary (and no observable pattern was present between values estimated using either of the regression models).

**Fig 4:**
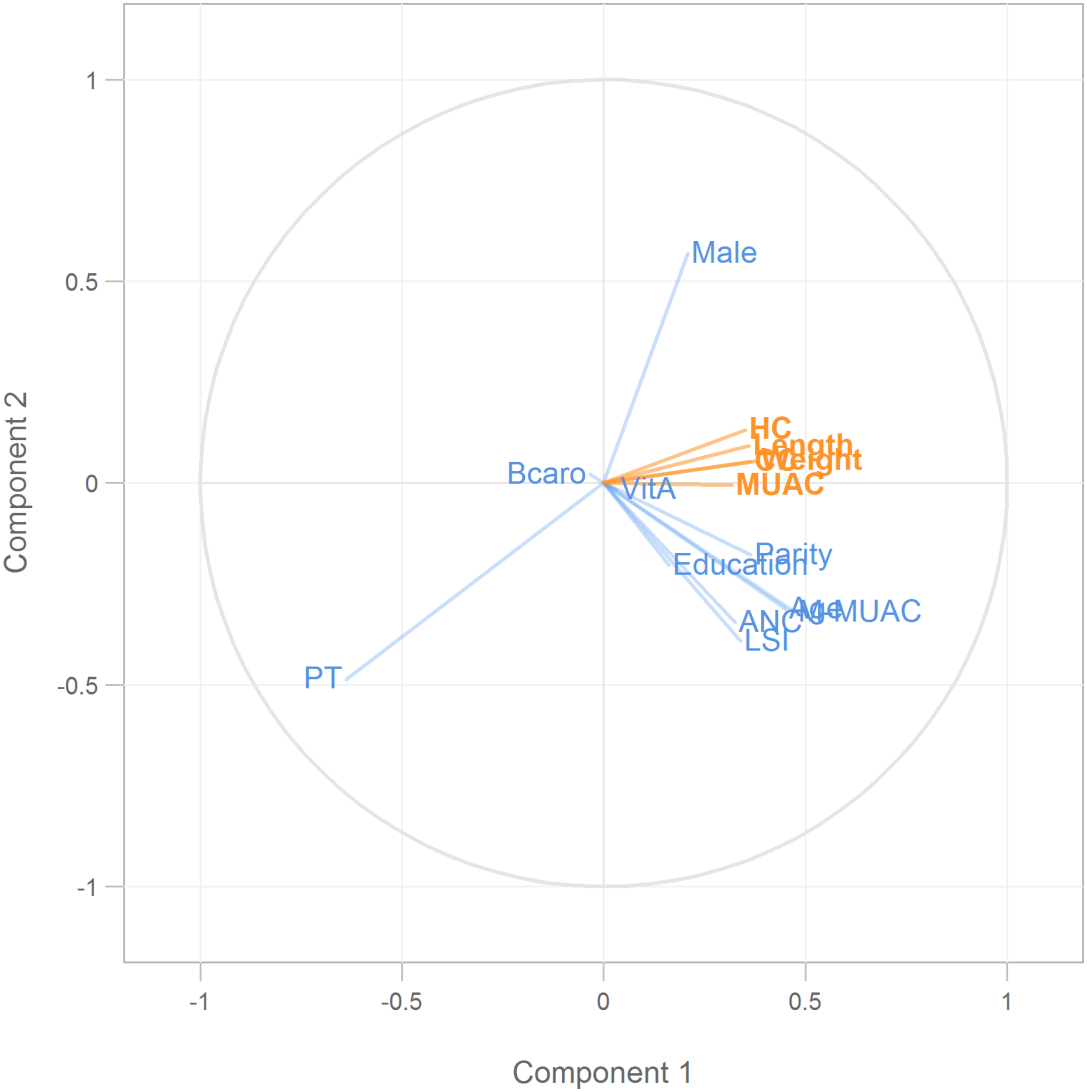
Correlation plot for the first two components. Orange color indicating the weights of dependent variables (birth size variables: weight, length, head (HC), chest (CC) and arm (MUAC) circumferences) and the blue color indicating the weights of the predictors (maternal and infant factors: maternal age, parity, education, MUAC, vitamin A sup. (VitA) and β-carotene sup. (Bcaro), living standard index (LSI), antenatal care visit (ANC), preterm delivery (PT), and infant’s sex (Male)).

The correlation plot of the variables for the first two components (Fig 4) depicted that maternal education, ANC visit, LSI, age, MUAC and parity were correlated and fell in the 4^th^ quadrant. Among them, age, MUAC and parity were close to each other and they were also closer to the birth size variables in the 1^st^ quadrant. Education, ANC visit, and LSI were very close to each other but were further from to the birth size variables compared to the prior cluster of maternal variables. On the other hand, preterm delivery alone fell in the 3^rd^ quadrant just opposite to the birth size variables and infant’s sex alone fell in the 1^st^ quadrant with the birth size variables. Maternal Vitamin A and β-carotene supplementation fell very close to the center indicating no effect on birth size. All the 5 birth size variables in the 1^st^ quadrant were clustered close to each other. Therefore, this figure demonstrated that preterm delivery was the most important predictor in the opposite direction, followed by infant’s gender, parity, age and MUAC.

**Fig 5:**
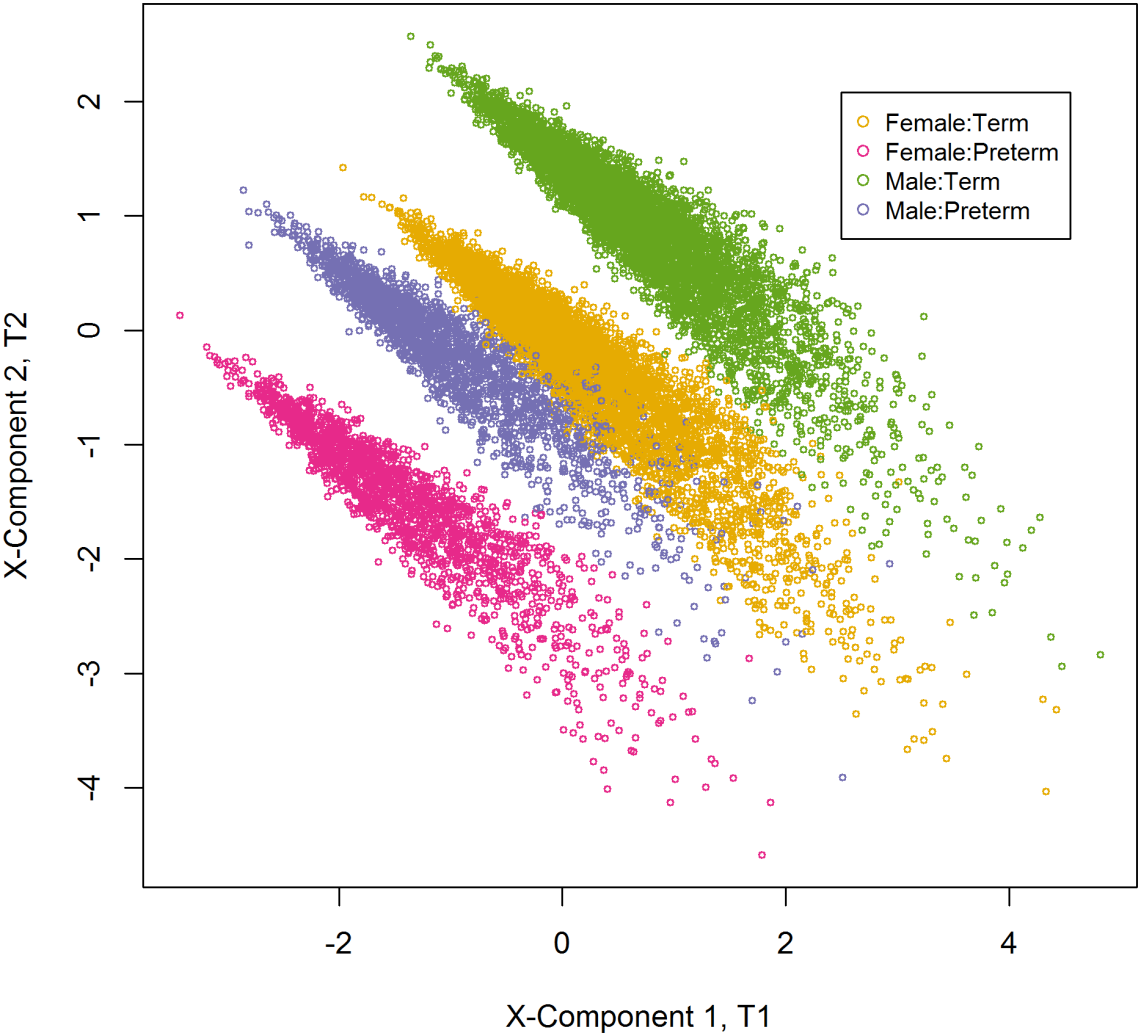
Scatter plot of the scores of first two PLS components of the predictors. Demonstrating 4 groups among the individuals participated in the study due the interaction effect of infant’s sex and preterm delivery.

The score plot of the 1^st^ and 2^nd^ PLS components of the predictors displayed four distinct groupings among the study participants due to the interaction effect of preterm delivery and infant’s sex (male:term, male:preterm, female:term and female:preterm) (Fig 5).

We observed that the magnitude of correlation between the predicted values using PCR and observed values of birth size was substantially increasing with increasing the number of components, however, the correlation was very poor with the first few components (Table 3). On the other hand, the magnitude of correlation between predicted values using PLS regression and the observed values of birth size did not meaningfully change with increasing the number of components. However, the explained variance of the predictors by the components of PLS regression was always lower than that of the PCR. Notably, the first 2 PLS components that were derived from maternal factors explained only ~26% of total variation of maternal factors had a predictive ability that was comparable to the PCR with 5 components that explained ~73% of total variation.

**Table 3:** Coefficients of Pearson’s correlation between the predicted values of birth size (by PLS and principal component regression) and observed values and the variance of the maternal and infant characteristics explained by the components of PLS and principal component regression analysis.

Infant’s size	PLS regression					Principal component regression				
	Comp-1	Comp-2	Comp-3	Comp-4	Comp-5	Comp-1	Comp-2	Comp-3	Comp-4	Comp-5
Correlation coefficients										
Weight	0.381	0.385	0.386	0.386	0.386	0.003	0.016	0.276	0.281	0.383
Length	0.360	0.372	0.372	0.372	0.372	0.007	0.014	0.240	0.243	0.365
MUAC	0.318	0.318	0.322	0.323	0.324	0.008	0.011	0.251	0.267	0.321
Head circumference	0.351	0.375	0.378	0.378	0.378	0.026	0.030	0.217	0.218	0.371
Chest circumference	0.364	0.368	0.372	0.373	0.373	0.002	0.012	0.257	0.272	0.368
Variance explained ($R_{x}^{2}$)^2^	12.59%	23.64%	37.89%	57.08%	62.84%	23.79%	38.82%	52.92%	63.14%	72.82%

^2^Vaciance explained by the components of maternal characteristics which were generated to model birth size and they were presented as cumulative from comp-1 to comp-5.

## Discussion

We conducted this study to assess the effect of maternal socio-demographic factors on newborn size at birth using PLS regression. Our study revealed that all the maternal variables examined, except vitamin A and β- carotene supplement receipt during pregnancy, were significantly associated with birth size. Preterm delivery had the greatest effect on birth size followed by infant sex, maternal parity, education, and age. In addition, PLS regression facilitated the finding of an interaction effect of preterm delivery and newborn sex on birth size, as revealed by a scatter plot of the first two components (Fig 5). PLS regression was more parsimonious than PCR and the ordinary least squares regression, as it required only two components compared to the five required in PCR regression, while ordinary least squares would have required the inclusion of all covariates. The PLS regression provided effect measures of the covariates with more stability and greater accuracy compared to the ordinary least squares regression.

In addition to birth weight, the combination of all birth size measurements captures more information than a single measurement in isolation; however, they are highly correlated. Head circumference, for instance, indicates the brain volume [30] and it may also provide important diagnostic and prognostic information like neurocognitive function [31], beyond that provided by birth weight alone. Therefore, it is expected that along with birth weight, other birth size measurements like length and head, chest and arm circumferences can provide more information associated with broader range of health outcomes like future growth, health and development. Hence, identification of maternal factors associated with newborn size using PLS regression has an important implication because PLS regression can simultaneously deal with multiple outcomes and collinearity among the covariates. A simulation study reported that in multiple regression settings, the correlation between covariates exceeding 0.35 has a greater impact on both the coefficients and their standard errors [32]. In case of collinearity, the effect of one variable may be confounded by other which cannot be fully extracted by general linear model [33]. Therefore, we need to be more conservative to account for collinearity in investigating the effect of individual risk factors on outcome. Despite the unbiasedness of the ordinary least squares regression coefficients, their standard error (SE) becomes larger with the degree of collinearity among covariates. This analysis also provided the evidence of having larger SE of the coefficients if the ordinary least squares regression was applied instead of PLS regression. However, PLS regression provides biased estimates, it provides a trade-off between bias and precision [34–36]. Moreover, the traditional ordinary least squares regression analysis with the adjustment of multiple confounders inadequately allocates the effect of covariates on outcome in presence of collinearity [33]. Therefore, PLS regression distributes the overall contribution of each of the maternal factors on birth size according to the correlations among covariates and outcomes. In this analysis it is obvious that the effect of the maternal variables with a higher level of collinearity on birth size obtained from PLS regression was substantially different from that of the ordinary least squares regression.

The PLS regression analysis suggested that maternal and infant factors age, parity, early pregnancy MUAC, LSI, maternal education, number of ANC visits and infant sex were significantly and positively associated with infant’s size at birth; however, preterm delivery had the greatest negative effect on birth size as anticipated. We did not find any effect of maternal vitamin A and β-carotene supplementation on infant’s size at birth and which is consistent with the result found by Christian et al. [37] and Kabir et al. [38] from the same population. This study also identified that preterm delivery and newborn sex had a significant interaction on size at birth which was also commensurate with the findings from a previous study where canonical correlation analysis was applied to the same dataset [38] and which was also consistent with other study [39]. The correlation plot of first two components of the maternal factors and infant’s size at birth indicated that maternal education, the number of ANC visits and the living standards index are correlated and maternal age and MUAC are also correlated which also provided evidence of collinearity existing among the maternal factors. Thus, we think the PLS regression was the appropriate method to use in this study.

PLS regression has the ability of handling multicollinearity and multiple dependent variables simultaneously. It has a proven ability to perform better than other regression methods, which are usually used to handle highly collinear data, such as stepwise multiple regression, PCR or model fitting techniques that apply Maximum Likelihood or Bayesian theory and in addition it equally performs in ideal situations [15,33,40]. The PLS method simultaneously predicts a set of dependent variables from a set of independent variables, instead of using separate regression models for each dependent variable. Typically in regression analysis, we look for a parsimonious model, i.e. the model that can predict the response variable at the desired level with as few predictors as possible. Because, however, R^2^ increases with an increasing number of predictors in the model, the variance of regression coefficients also increases. Thus, it is statistically advantageous to have a reduced number of explanatory variables in a given model. Compared to PCR, PLS regression requires a smaller number of components resulting in more stable estimates of regression coefficients. In this analysis, PLS regression required only two components but PCR required five. Despite the fact that PLS components explained a much lower variance (~24%) of the maternal and infant factors, it had more predictive power over the PCR where components explained ~73% variance. This is because, in PLS regression, a pair of components is chosen from the dependent and independent variables so that they are closest to each other; however, in PCR, a component is chosen from the independent variables which capture most of the variability without accounting for how close it is to the dependent variables. Criteria that give penalties on the number of variables, like Akaike Information Criterion (AIC), or those where model performance is evaluated, like the Mallows Cp criteria, also encourage the need to include more variables than the PLS method [28]. This aspect of efficiency provides a basis for obtaining better prediction without necessarily needing to explain a large amount of variability of the independent variables by the extracted components in PLS regression.

## Conclusions

PLS regression was a better choice to analyze this data. It needed only two components, while PCR, the most commonly used method, required 5 components to have similar predictive power. The coefficients obtained from PLS regression were more stable and accurate than that of the general linear model. Using PLS regression, the maternal factors identified as the significant predictors of newborn size at birth were commensurate with other studies [41–49]. So, PLS regression has the promising potential as a multivariate regression method in public health research to address the innate complexity of interactions and biological pathways between variables.

## Supporting information

S1 Dataset Analytic dataset. (XLSX)

S1 File Code book of the analytic data set. (DOCX)

S1 Table Comparison between coefficients estimated from PLS and ordinary linear regression. (DOCX)
