# Supplementary material for: Identifying maternal and infant factors associated with newborn size in rural Bangladesh by partial least squares (PLS) regression analysis
Source: PLoS One. 2017 Dec 20;12(12):e0189677. doi: 10.1371/journal.pone.0189677 (PMC5738092; doi:10.1371/journal.pone.0189677)
Supplement: S1 File — (DOCX) [file pone.0189677.s002.docx]

Code book

| Variable Name | Description | Code |
| --- | --- | --- |
| compid | Unique ID |  |
| Weight | Infant’s weight at birth, kg | Exact value |
| Length | Infant’s length at birth, cm | Exact value |
| MUAC | Infant’s mid-upper arm circumference (MUAC) at birth, cm | Exact value |
| HC | Infant’s head circumference at birth, cm | Exact value |
| CC | Infant’s chest circumference at birth, cm | Exact value |
| Parity | Maternal parity at recruitment | Exact value |
| Age | Maternal age, yr | Exact value |
| M-MUAC | Maternal MUAC, cm | Exact value |
| LSI | Living standard index | Exact value |
| Education | Maternal education, years of education | Exact value |
| ANC | No of antenatal care visit | Exact value |
| Male | Male infant | 1=Male  0=Female |
| PT | Preterm delivery | 1=Preterm (gestational age <37 wks)  o=Term (gestational age ≥37 wks) |
| VitA | Received vitamin A supplementation | 1=Received vitamin A  0=Received other |
| Bcaro | Received β-carotene supplementation | 1=Received β-carotene  0= Received other |
