# Supplementary material for: Identifying maternal and infant factors associated with newborn size in rural Bangladesh by partial least squares (PLS) regression analysis
Source: PLoS One. 2017 Dec 20;12(12):e0189677. doi: 10.1371/journal.pone.0189677 (PMC5738092; doi:10.1371/journal.pone.0189677)
Supplement: S1 Table — (DOCX) [file pone.0189677.s003.docx]

S1 Table: Comparison between coefficients estimated from PLS and ordinary linear regression (LR)

|  | Weight  β (SE) | | Length  β (SE) | | MUAC  β (SE) | | HC  β (SE) | | CC  β (SE) | |
| --- | --- | --- | --- | --- | --- | --- | --- | --- | --- | --- |
|  | PLSR | LR | PLSR | LR | PLSR | LR |  | LR | PLSR | LR |
| **Age** | **0.103 (0.005)** | **0.069 (0.008)** | **0.086 (0.006)** | **0.077 (0.013)** | **0.100 (0.004)** | **0.054**  **(0.013)** | **0.072 (0.009)** | **0.046 (0.012)** | **0.098 (0.005)** | **0.068 (0.013)** |
| **Parity** | **0.106 (0.005)** | **0.145 (0.013)** | **0.097 (0.006)** | **0.114 (0.013)** | **0.092 (0.004)** | **0.139**  **(0.013)** | **0.092 (0.006)** | **0.114 (0.013)** | **0.102 (0.005)** | **0.141 (0.013)** |
| Early pregnancy MUAC | 0.114 (0.006) | 0.124 (0.008) | 0.098 (0.008) | 0.083 (0.008) | 0.108 (0.005) | 0.119 (0.008) | 0.085 (0.006) | 0.089 (0.008) | 0.109 (0.007) | 0.106 (0.008) |
| Education | 0.034 (0.005) | 0.036 (0.010) | 0.028 (0.004) | 0.036 (0.010) | 0.034 (0.005) | 0.044 (0.010) | 0.024 (0.004) | 0.042 (0.010) | 0.033 (0.005) | 0.042 (0.010) |
| LSI | 0.063 (0.005) | 0.054 (0.010) | 0.047 (0.003) | 0.049 (0.010) | 0.067 (0.003) | 0.049 (0.010) | 0.034 (0.006) | 0.042 (0.010) | 0.060 (0.004) | 0.045 (0.010) |
| Preterm | -0.266 (0.004) | -0.269 (0.008) | -0.274 (0.007) | -0.279 (0.008) | -0.193 (0.006) | -0.220  (0.008) | -0.290 (0.005) | -0.270 (0.008) | -0.254 (0.007) | -0.281 (0.008) |
| No of ANC visit | 0.063 (0.004) | 0.061 (0.008) | 0.049 (0.004) | 0.053 (0.008) | 0.066 (0.005) | 0.052  (0.008) | 0.037 (0.007) | 0.045 (0.008) | 0.060 (0.005) | 0.048 (0.008) |
| Vitamin A sup | 0.004 (0.006) | -0.003 (0.009) | 0.003 (0.005) | 0.003 (0.009) | 0.004 (0.005) | -0.009  (0.009) | 0.003 (0.006) | 0.009 (0.009) | 0.004 (0.005) | -0.009 (0.009) |
| β-carotene sup | -0.009 (0.006) | -0.018 (0.018) | -0.008 (0.005 | -0.007 (0.009) | -0.008 (0.005) | -0.015  (0.009) | -0.008 (0.006) | -0.003 (0.009) | -0.009 (0.005) | -0.020 (0.009) |
| Male infant | 0.118 (0.006) | 0.109 (0.008) | 0.135 (0.006) | 0.128 (0.008) | 0.068 (0.005) | 0.024  (0.008) | 0.156 (0.009) | 0.185 (0.008) | 0.113 (0.006) | 0.070 (0.008) |
| **R^2^** | **0.15** | **0.15** | **0.14** | **0.14** | **0.10** | **0.10** | **0.14** | **0.14** | **0.14** | **0.14** |
